# Do Running and Strength Exercises Reduce Daily Muscle Inactivity Time?

**DOI:** 10.3934/publichealth.2016.4.702

**Published:** 2016-09-06

**Authors:** Taija Finni, Marja Uusi-Vähälä, Arto J. Pesola, Ritva S. Taipale

**Affiliations:** 1 Neuromuscular Research Center, Department of Biology of Physical Activity, University of Jyväskylä, Jyväskylä, Finland; 2 Kajaani University of Applied Sciences, Kajaani, Finland

**Keywords:** muscle inactivity, muscle activity, physical activity, strength, endurance, combined training, daily sedentary time

## Abstract

Understanding how a specific exercise changes daily activity patterns is important when designing physical activity interventions. We examined the effects of strength and interval running exercise sessions on daily activity patterns using recordings of quadriceps and hamstring muscle electromyographic (EMG) activity and inactivity. Five male and five female subjects taking part in a 10-week training programme containing both strength and interval running training sessions were measured for daily muscle EMG activities during three days: on a strength day, an interval running day, and a day without exercise. EMG was measured using textile electrodes embedded into sport shorts that were worn 9.1 ± 1.4 hours/day and results are given as % of recording time. During the total measurement time the muscles were inactive 55 ± 26%, 53 ± 30% and 71 ± 12% during strength training day, interval running day, and day without exercise (n.s.). When compared to the day without exercise, the change in muscle inactivity correlated negatively with change in light muscle activity in strength (r = -0.971, *p* < 0.001) and interval running days (r = -0.965, *p* < 0.001). While interval running exercise bout induced a more systematic decrease in muscle inactivity time (from 62 ± 15% to 6 ± 6%, *p* < 0.001), reductions in muscle inactivity in response to strength exercise were highly individual (range 5–70 pp) despite the same training programme. Strength, but not running exercise bout, increased muscle activity levels occurring above 50% MVC (*p* < 0.05) when compared to a similar period without exercise. The effect of strength exercise bout on total daily recording time increased the EMG amplitudes across the entire intensity spectrum. While strength and interval running exercise are effective in increasing muscle moderate-to-vigorous activity when compared to a similar period without exercise, it comprises only a small part of the day and does not seem to have a systematic effect neither to reduce nor induce compensatory increase in the daily muscle inactivity that is highly heterogeneous between individuals.

## 1. Introduction

The entire spectrum of physical activity (PA) from light to vigorous PA is important for maintaining and improving health, functional capacity and fitness [1]. On the other end of the spectrum, sedentary behaviour is associated with adverse health outcomes independent of participation in moderate-to-vigorous physical activity (MVPA) [2],[3] suggesting that some of the health effects of light intensity physical activity can be attributed to reduced sedentary time.

By definition, sedentary behaviour refers to energy expenditure below 1.5 METs and a sitting or reclining posture [4]. In the field of sedentary behaviour, however, the driving hypothesis of inactivity physiology is not linked to the posture but to the absence of muscular activity which causes the detrimental physiological processes of sedentary time [5]. These processes may be short-circuited by frequent activity of muscles, which has been associated with better lipid profiles, for example [6]. Because the presence of muscle activity is included inherently to the definition of PA [7], electromyographic recordings of muscle activity (EMG) may help us to understand the mechanisms how the different aspects of physical activity might mitigate the health hazards of sedentary time.

Depending on which of the domains are included in the exchange between physical activity and sedentary time, the efficacy of a given intervention may either increase or decrease. An exercise bout typically increases MVPA and this increase must be compensated by changes in other domains of PA. In a group of relatively active individuals we previously showed that while exercise for fitness increased muscle MVPA, it did not significantly reduce muscle inactivity time when measured using EMG from large locomotor muscles [8]. The observation that MVPA is not systematically compensated for by a reduction in either light activity or sedentary time suggests that the interaction between physical activity and sedentary behaviour can be very individual.

The compensatory effects of MVPA might depend on the type of exercise performed. Current PA guidelines promote both aerobic and resistance exercises, because the different activation patterns result in distinct physiological adaptations and complementary health benefits [9]. While running training contains typically repetitive cyclic loadings with a given intensity, strength exercises can have brief and very high intensity loadings with considerable rest periods between cycles. Consequently, the energy expenditure is higher during aerobic than resistance exercise [10], but short bouts of intense exercise such as occur in strength training promote neuromuscular function and maintenance of muscle mass [11] without major effects on energy expenditure [12]. However, the physiological adaptations are not limited to the training session. Studies looking at the interplay between the different PA intensity domains in response to a training programme have shown that an aerobic training day is associated with a decline in non-exercise PA without effects on sedentary time [12]–[14], whereas a strength training day is associated with increased non-exercise activity on days without exercise [15]. Thus, understanding of the eventual dose of PA and the resulting physiological adaptations warrants a detailed analysis of the compensatory mechanisms of different modalities of PA across several days.

To study the compensatory effects of different training modes it is important to quantify the intensity, frequency, duration and lack of physical activity accurately. Objectively measured sedentary behaviour is typically assessed using hip or waist mounted accelerometers and reported as total sedentary time identified as <100 counts per minute [16]. In some studies thigh mounted accelerometers (e.g. [17]) have been used to distinguish sitting from quiet standing in order to have a more reliable measure of sedentary time as per the definition [4]. However, accelerometer-derived sedentary time may underestimate physical activity during short fidgeting-like activities [18], cycling [19], uphill and downhill gait [20] or strength-training [21]. Moreover, accelerometers are incapable of measuring the intensity of PA relative to an individual's physiological capacity during static or dynamic conditions such as occur during strength training. To overcome the inherent problems of accelerometry our group has established the use of EMG to accurately detect daily muscular inactivity and activity patterns of an individual. Provided that the benefits of reducing sedentary time have been proposed to be caused by increased muscle activity, but mediated through reallocation to different muscle activity intensities, it is important to measure the exposure of a treatment in this outcome on a total muscle activity spectrum. Because of directly measuring muscle activity at a very short time-window, EMG can detect small muscle activities also during sedentary behaviour [22] and allow high intensities to be quantified with detail. EMG amplitude reflects the actual contraction intensity of skeletal muscles, which is the primary cause for increased energy expenditure and, consequently, is also a valid measure for PA level [20]. Because of these inherent differences the EMG-derived muscle inactivity may not correspond with accelerometer-derived sedentary time, but give new insights to the field with a measure that is at the heart of the sedentary behaviour epidemic [5].

Therefore, in the present study we used EMG when examining the effects of strength exercise (STR) and interval running exercise (RUN) on muscle activity levels and muscle inactivity during the specific exercise bouts, as well as during the whole day. It was hypothesised that STR and RUN exercises would have distinct activity patterns that differ from those occurring during a normal day without purposeful exercise (REST). We also compared the effects of STR and RUN on the distribution of activity levels in order to investigate if and how PA compensation occurs.

## 2. Materials and Methods

### 2.1. Design

A repeated measures design was used. The subjects engaged in a 10-week training programme consisting of both strength training and interval running sessions on separate days. The study was approved by the Ethics Committee of the University of Jyväskylä and the subjects signed an informed consent prior to any measurements.

### 2.2. Recruitment

Healthy, recreationally physically active young men and women were recruited by advertisements online and in the local newspaper. Inclusion criteria were 18–40 years of age, healthy, and physically active young men and women who participated in primarily endurance exercise and team sports (like floorball and football/soccer) but also had some informal strength training experience. Exclusion criteria were BMI > 30 kg/m^2^, any diseases, musculoskeletal or cardiac problems, or medications that would preclude a subject's ability to perform resistance and endurance training and testing.

A total of 25 volunteers signed up for the study. After explaining the protocol 6 subjects dropped out due to personal/scheduling reasons. Nineteen subjects began the study and were measured for baseline. Sufficient data with adequate quality was obtained from 10 subjects (5 women, 5 men, age 29 ± 5 yrs; height 173.2 ± 8.8 cm; body mass 71.0 ± 12.0 kg) who fulfilled the criteria of having three days (STR, RUN and REST) of muscle activity recordings. The nine subjects that were not included in the analysis had missed a recording day (N = 6) or had insufficient data for analysis (N = 3).

### 2.3. Protocol

Prior to participating in this study, subject's resting electrocardiography and health questionnaires were screened and approved by a medical doctor. Subjects were measured in the laboratory before and after the 10-week intervention for height and body mass, lower body maximal strength and endurance performance. Day-long EMG-measurements were done on three days, one with a strength training session (STR), one with an interval running session (RUN) and one without any exercise session (REST) in random order depending on availability of the subjects for the EMG-measurements and also depending on the availability of the equipment, which were limited to two simultaneous measurements. The EMG recordings began between 7 and 9 a.m. so that within each individual the beginning time was as consistent as possible during the three days. The first two weeks of intervention was allowed for familiarization after which the daily measurements began. For a given subject the measurements for STR and RUN were completed within two weeks (mean 3.7 days in between) and REST not more than four weeks away from STR or RUN.

### 2.4. Intervention

The 10-week training programme consisted of interval running training twice a week and strength training twice a week. The training programme was designed according to the current PA recommendations with known effects on improving performance [23]. Combined training was designed to have a realistic programme applicable for recreationally active persons and that was modified from previous research from our lab [24],[25]. Training was completed either in the morning (between 7 and 9 a.m.) or afternoon (between 4 and 7 p.m.) during workdays depending on the subjects' personal schedules but within the subject the schedule was consistent. The strength training sessions were supervised but some running sessions were performed individually with subjects monitoring their heart rates and keeping log on training duration.

*Strength training sessions* consisted of a mixture of maximal and explosive strength training for both the lower and upper extremities. Exercises for the lower extremities included half-squat, squat-jump, leg-press, knee flexion, calf-raises, and calf-jumps as well plyometric jumps. The primary exercise for the upper extremities was the bench-press. Traditional core exercises such as plank, back extension and oblique exercises were also included as they are a typical part of training programs for all athletes. Maximal and explosive strength training are known to increase muscle strength and induce positive changes in body composition as well as metabolism [24]. The strength training loads were individually tailored according to % RM while the exercises in the strength training program were standard for all subjects throughout the study.

*Interval running training sessions* included 4 × 4 min running intervals at approximately 90% of heart rate max (measured on the treadmill) as well as 3 × 3 × 100 m sprints with 4 min breaks to include more neural stimulus. We chose the 4 × 4 min running intervals with 4 minute break based on [23] in which these intervals were considered as effective as multiple high-intensity sprints in increasing aerobic capacity. Performed twice a week, these intervals have been shown to increase endurance capacity.

### 2.5. Measurements

Maximal dynamic bilateral concentric strength (1RM) was measured with leg press (David Sports Ltd., Helsinki, Finland). Prior to attempting 1RM, subjects completed warm-up lifts consisting of 5 × ∼70% 1RM, 2 × ∼80–85% 1RM and 1× ∼90–95% of estimated 1RM, with one minute of rest between sets. Following this warm-up, no more than 5 attempts to reach 1RM were made. The leg extension action started from a knee angle of ∼60 degrees. Subjects were instructed to grasp handles located by the seat of the dynamometer and to keep constant contact with the seat and backrest during leg extension to a full range of motion (180 degrees). Verbal encouragement was given to promote maximal effort. The greatest weight that the subject could successfully lift (knees fully extended) to the accuracy of 2.5 kilograms was accepted as 1RM.

Endurance performance was measured by performing a 3000 m time-trial, timed with a stop watch, which was run on a 200-m indoor track approximately one week before the training period and approximately one to two weeks after the training period.

Assessment of Daily Muscle EMG Activity

In the morning the subjects put on the EMG-shorts (Mbody, Myontec Ltd., Kuopio, Finland). The size of the shorts was selected to fit the subject tightly in order to sustain proper skin-electrode contact. The shorts had a bipolar electrode configuration for recording muscle activity from the left and right quadriceps and hamstring muscle groups. Conductivity enhancing gel (Redux Electrolyte Créme, Parker Laboratories Inc., Fairfield NJ, USA) was applied to the large textile electrodes that provided a global measure of muscle activity of the main locomotor muscles. The EMG signal from the four muscle groups was stored into a 52 g module located at the waistband. Raw EMG signal, recorded with 1000 Hz sampling rate was band pass filtered with 50 Hz–200 Hz (-3 dB), rectified and averaged over 100 ms non-overlapping periods before storage in the module. This procedure allowed recording periods of over 12 h. For further details of the EMG methodology and reliability please see [26]–[28].

In the morning the subjects were asked to perform the following tasks: 2 minutes of sitting and 2 minutes of standing quietly as well as 1 minute of walking at a self-selected speed in a long corridor. For a reliability check, some subjects also repeated this in the evening before finishing the measurement.

Maximal isometric voluntary contractions (MVC) of knee extensors and flexors with a knee joint ankle of 107 degrees (David 200, David Health Solutions, Helsinki, Finland) were measured and the best result of 3 trials was taken to represent MVC and was used for EMG normalization. In the case that the 3^rd^ attempt increased more than 5% compared to the previous attempt, a 4^th^ attempt was made to get the best possible result.

The subjects were asked to live their day as usual and according to their normal schedule including the training schedule (STR, RUN or REST). EMG measurements were done after the subjects were familiarized to the training during weeks 2–10 of the intervention. Availability of the two recording modules was a limiting factor for scheduling these assessments.

The subjects were instructed to remove the EMG shorts when taking a shower and marking other possible interruptions (such as a visit to the toilet) by pressing a button on the module that created a marker in the data. The daily measurements ended in the evening before bedtime at the latest, but typically after the evening training session.

### 2.6. EMG Analysis

The data was visually assessed and, if necessary, corrected for artifacts with established procedures [27],[28]. Brief, non-physiological signals (defined as a peak where the difference in the amplitude of two consecutive data points was >150 μV) were replaced with a median value from 10 data points prior to and following the artifact. With the help of markers inserted by the subjects when the shorts were removed for any reason, the resulting artifacts were removed.

Maximal EMG values (EMG_MVC_) were taken as an average from a one second period from the middle of the MVC where the signal was most consistent. The EMG signal from each of the four muscle groups was normalized to the corresponding EMG_MVC_ value and are reported as percentage of EMG_MVC_. The normalizing procedure was done for each day separately. After normalizing each of the four channels, they were averaged to create a mean thigh muscle EMG. Thus, the results reflect overall inactivity and activity periods and are not specific to a single muscle. The EMG data was corrected for baseline drift by using a moving filter on a 5 minute window with a custom made Matlab algorithm (The MathWorks Inc, version 7.11.0.587) [28].

From the tasks performed each morning/evening, the average EMG values for sitting, standing and walking were analyzed using time a window up to 2 minutes. Muscle inactivity was defined as EMG amplitude below 90% of that during standing [6],[27],[28]. Light muscle activity contained EMG intensities above inactivity but below that during preferred walking speed. Mean EMG during preferred walking was on average 12.1 ± 9.9% MVC for quadriceps and 4.3 ± 5.6% MVC for hamstrings in all participants. The overall average for both muscle groups was 7.8 ± 8.9% MVC that corresponded very close to 3 METs as reported previously [27]. EMG amplitudes above this were considered as muscle MVPA.

The durations of muscle inactivity, light and MVPA were calculated using custom made Matlab script and reported as % of total time. The data was analyzed for full recording time (9.1 ± 1.4 h) and separately for a 50 minute time window representing RUN, STR and REST periods. The 50 min REST period was taken from a day without training during the same time of day when the participant executed the training. EMG variables calculated were average amplitude, average burst amplitude (average amplitude for activity above the inactivity threshold), inactivity time, light activity time, MVPA time, and sum of the five longest inactivity periods (long inactivity periods). EMG amplitude distribution was further examined by analyzing the proportion of different amplitude levels (0–5%, 5–10%, 10–20%, 20–30%, 30–40%, 40–50%, 50–60%, 60–70%, 70–80% and ≥80% of MVC). The difference (REST-STR and REST-RUN) in selected variables between the exercise sessions and during the total recording time were calculated and the results are given as percentage points (pp).

### 2.7. Statistics

Data was first checked for normality. Repeated measures analysis of variance (ANOVA) was applied for assessing the difference in variables between STR, RUN and REST. Contrasts comparing STR and RUN to REST were used to examine the effects of training session on physical activity patterns during the 50 min period and whole day. Furthermore, differences between STR and RUN were examined using contrasts. Differences in EMG variables between the 50 min period and whole day were compared using paired t-test. Effects of the training intervention on strength and endurance capacity were tested by using paired t-test. Pearson's product moment correlation coefficient was calculated between differences in REST-STR and REST-RUN to examine the occurrence of tradeoff between muscle activity intensities. Results are reported as means and standard deviations. The level of significance was set to *p* < 0.05.

## 3. Results

Overall, the 10-week training programme increased leg extension strength by 9.3% (*p* = 0.008) and the 3000 m time-trial improved by 4.0% (*p* = 0.014). On the three days of measurements the recording times were not significantly different (9.4 ± 1.0 h in STR, 9.5 ± 1.7 h in RUN and 8.4 ± 1.3 h in REST) and the recordings were done approximately at the same time of day on each three days. On average, the maximum difference in the start of recording between the three days was 45 minutes (range 5–105 min).

**Figure 1.**
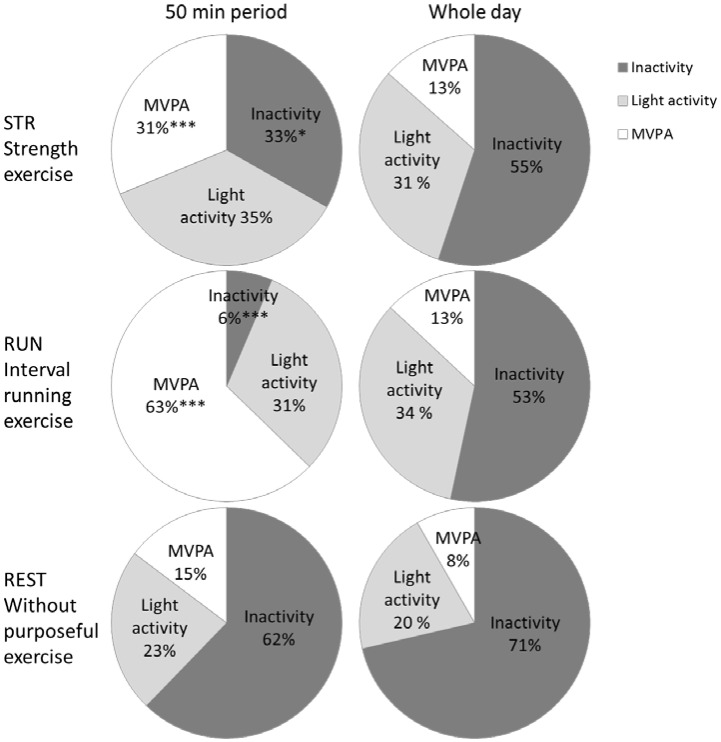
The proportions of muscle inactivity, light muscle activity and muscle MVPA time during the 50 min period and whole day. Distribution of muscle inactivity (corresponding to EMG amplitude below 90% of that during standing), light activity (corresponding to that above inactivity but below 3 METs) and moderate to vigorous muscle activity (MVPA, corresponding to >3 METs) time during strength exercise, interval running exercise and without purposeful exercise (left panel). In right panel, the distributions are shown during the entire recording period (whole day). Significant differences in distribution were found between the 50 min period and whole day in STR and RUN but not in REST. Different from the whole day at * *p* < 0.05, ** *p* < 0.01 and *** *p* < 0.001.

The proportions of muscle inactivity, light muscle activity and muscle MVPA time during the 50 min period and whole day are shown in Figure 1. As compared to the distribution of muscle activities during the entire day, both STR and RUN exercise bouts contained different proportions of muscle inactivity and MVPA. In REST, there were no significant differences between the 50 minute session and the total measurement time reflecting that the distribution of activity intensities was comparable.

### 3.1. Comparison of the 50 min Periods

Table 1 shows the mean values of EMG amplitude variables. When STR and RUN exercise sessions were compared to REST, the average EMG amplitude and average burst amplitude were significantly higher during STR and RUN than during REST. There was less muscle inactivity and more muscle MVPA during STR and RUN than in the REST period. Also light muscle activity in STR was significantly greater than during the REST period. The sum of the five longest inactivity periods during STR and RUN were shorter than during REST.

When STR was compared to RUN, average EMG and average burst EMG amplitude were smaller, and muscle MVPA time was shorter in STR as compared to RUN. However, long inactivity periods and total inactivity time were longer during STR than in RUN (Table 1).

Comparison of the distribution of different EMG amplitude levels in Table 2 shows how the amplitudes below 5% MVC were reduced and a proportion of the higher amplitudes increased in response to STR and RUN. As compared to REST, STR increased also the activity levels above 50% MVC while RUN did not. However, the differences between STR and RUN were significant only at intensities below 30% MVC (Table 2).

**Figure 2.**
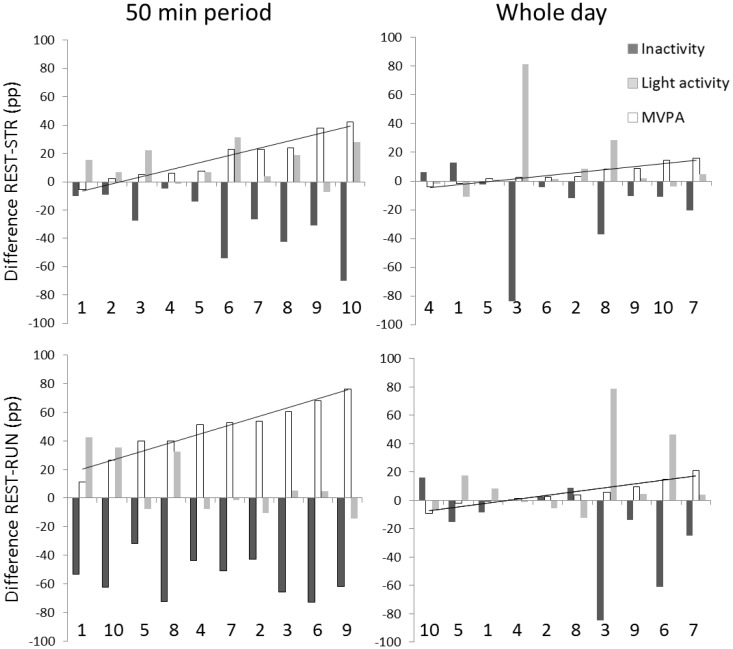
The differences in muscle inactivity, light muscle activity, and muscle MVPA time calculated as REST-STR and REST-RUN. Differences between the 50 minute time period (left panel) or the day (right panel) without exercise (REST) and with strength exercise (STR, upper panel) or with interval running exercise (RUN, lower panel) shown as percentage points (pp) for muscle inactivity time (dark gray), muscle light activity time (light gray) and moderate-to-vigorous muscle activity time (MVPA, white bars) for each ten individuals. Individuals are numbered from 1 to 10 and shown in the abscissa. Data is organized from smallest to largest difference in MVPA where the trend line is also shown.

Figure 2 shows the differences in muscle inactivity, light muscle activity, and muscle MVPA time calculated as REST-STR and REST-RUN. In the 50 minute STR period, the change in muscle inactivity time correlated negatively with the change in muscle MVPA (r = -0.804, *p* = 0.005) and light muscle activity (r = -0.673, *p* = 0.033). During RUN, on the other hand, the change in muscle inactivity was not associated with changes in muscle activity. However, the change in muscle MVPA during RUN correlated negatively with change in light muscle activity (r = -0.778, *p* = 0.008).

### 3.2. Comparison of the Total Measurement Time

During the total measurement time, average EMG amplitude and average burst amplitude were greater during STR than during REST (Table 1). These increases in muscle activity took place over the entire intensity spectrum. Shown in Table 2, nearly all intensity levels in STR were significantly different from REST. The amplitudes below 5% MVC were reduced and above 5% MVC they were increased. On the contrary, the interval running exercise did not alter statistically significantly the distribution of daily muscle activity intensities when compared to REST. Furthermore, there were no significant differences between the STR and RUN days.

Figure 2 shows that the differences in muscle inactivity, light muscle activity, and muscle MVPA time between REST-STR and REST-RUN during the whole day were highly individual and that the differences in MVPA were within the range from -9 to 21 pp. However, the differences in light activity and inactivity correlated significantly in both STR (r = -0.971, *p* < 0.001) and RUN days (r = -0.965, *p* < 0.001).

**Table 1.** EMG amplitude characteristics during strength exercise (STR), interval running exercise (RUN) and without purposeful exercise (REST).

	50 min period				Whole day		
	STR		RUN	REST	STR	RUN	REST
**aEMG (% MVC)**	6.4 ± 2.3 ***	^#^	10.6 ± 4.5 ***	2.6 ± 1.5	2.8 ± 1.3 *	3.0 ± 1.8	1.8 ± 0.7
**Average burst EMG (% MVC)**	9.1 ± 2.4 ***	^#^	11.1 ± 4.3 **	5.3 ± 2.6	5.9 ± 2.2 **	5.5 ± 2.2	4.7 ± 1.7
**Long muscle inactivity periods (s)**	2.3 ± 1.6 **	^##^	0.4 ± 0.4 **	8.3 ± 5.6	22.7 ± 15	25 ± 16.8	36.6 ± 18.5
**Muscle inactivity (% recording time)**	33.2 ± 14.3 **	^###^	6.4 ± 5.7 ***	62.2 ± 15.2	55.1 ± 26.0	53.4 ± 29.8	71.4 ± 12.2
**Light muscle activity (% recording time)**	35.5 ± 15.4 *		30.9 ± 18.7	23.0 ± 11.0	31.4 ± 27.3	33.6 ± 30.4	20.3 ± 11.3
**Moderate-to-vigorous muscle activity (% recording time)**	31.2 ± 15.5 **	^#^	62.7 ± 21.7 ***	14.7 ± 11.8	13.4 ± 9.3 *	13.0 ± 9.1	8.3 ± 5.0

Comparisons were made using contrasts within the 50 minute bouts and the entire recording period (whole day). Muscle inactivity corresponds to EMG amplitude below 90% of that during standing, light muscle activity corresponds to <3 METs and moderate-to-vigorous muscle activity to that above 3 METs. Significant differences to REST at *p < 0.05, **p < 0.01, ***p < 0.001. Significant differences between STR and RUN at ^#^ *p* < 0.05, ^##^ *p* < 0.01, ^###^ *p* < 0.001.

**Table 2.** Time spent at different EMG intensities during strength training (STR), interval running (RUN) and without purposeful exercise (REST) expressed as a percentage of the recording time.

EMG amplitude (% MVC)	50 min period				Whole day		
	STR		RUN	REST	STR	RUN	REST
**0–5%**	69.1 ± 15.0 **	^##^	37.4 ± 20.9 ***	86.5 ± 10.7	85.7 ± 10.3 *	86.7 ± 10.3	91.9 ± 5.2
**5–10%**	15.6 ± 8.1 *	^#^	24.9 ± 6.4 ***	7.5 ± 5.4	8.6 ± 6.3 *	6.8 ± 3.9	5.2 ± 3.4
**10–20%**	8.3 ± 5.1	^##^	23.8 ± 12.5 **	4.7 ± 4.9	4.2 ± 3.6 *	4.5 ± 4.5	2.3 ± 1.7
**20–30%**	2.7 ± 1.3 *	^#^	9.0 ± 7.1 **	0.9 ± 1.2	0.8 ± 0.7	1.3 ± 1.3	0.4 ± 0.3
**30–40%**	1.5 ± 0.5 ***		2.9 ± 2.4 **	0.3 ± 0.4	0.3 ± 0.2 *	0.4 ± 0.5	0.1 ± 0.1
**40–50%**	1.0 ± 0.3 ***		0.9 ± 0.9 **	0.1 ± 0.1	0.1 ± 0.1 **	0.2 ± 0.2	0 ± 0
**50–60%**	0.7 ± 0.3 ***		0.4 ± 0.6	0 ± 0	0.4 ± 0.6	0.1 ± 0.1	0 ± 0
**60–70%**	0.4 ± 0.3 ***		0.2 ± 0.5	0 ± 0	0.1 ± 0.0 *	0.0 ± 0.1	0 ± 0
**70–80%**	0.3 ± 0.2 **		0.2 ± 0.4	0 ± 0	0.0 ± 0.0 *	0.0 ± 0.1	0 ± 0
**≥80%**	0.5 ± 0.6 *		0.3 ± 0.9	0 ± 0	0.0 ± 0.1 *	0.0 ± 0.0	0 ± 0

Comparisons were made using contrasts within the 50 minute bouts and the entire recording period. Significant differences to REST at * *p* < 0.05, ** *p* < 0.01, *** *p* < 0.001. Significant differences between STR and RUN at ^#^ *p* < 0.05, ^##^ *p* < 0.01.

## 4. Discussion

This study measured thigh muscle EMG activity during strength exercise and interval running exercise as well as during equivalent period without exercise to examine the change in muscle inactivity and activity patterns during the specific exercise bout as well as during the whole day. A session of either strength or interval running training was found to reduce muscle inactivity and increase muscle activity when compared to a similar period of a day without exercise. When the whole day was considered, only strength exercise had a significant effect and increased the overall EMG amplitude and moderate-to-vigorous muscle activity amplitude in particular. While these were the general trends, redistribution of EMG intensities in response to exercise was highly individual. This was evident from correlations showing that the subjects who decreased the daily muscle inactivity the most, reallocated this mostly to the light muscle activity and subjects who had only small decreases in inactivity had also small increases in light muscle activity. This was true for both strength and running exercise days. The results of this study imply that although the muscle activity patterns changed significantly during the training, neither the strength nor the endurance training had systematic effects on muscle inactivity time outside of the training sessions. Therefore, reduced muscle inactivity during training does not decrease total daily muscle inactivity, but is neither compensated by increased muscle inactivity in a group of young healthy adults.

Across the waking hours, there is time for both sedentary time and MVPA. The results of this study support the view from observational studies, where the sedentary time and moderate-to-vigorous activity patterns remain poorly correlated with each other [17],[29]. Similarly, workplace interventions targeting increased PA do not reduce self-reported sedentary time [30], and those targeting sedentary time do not increase MVPA [31]. However, the participants in an intervention study designed to increase MVPA obtained the change by reducing their sedentary behaviour [32], but other MVPA-targeted intervention studies have found that increases in PA may occur at the expense of non-exercise PA [13],[14]. The present results suggest that these reallocation mechanisms can be very individual. An exercise bout did not decrease muscle inactivity time significantly although the mean inactivity time decreased about 15 pp on a day of training. The fact that this large difference was not significant can be allocated to large inter-individual variation in the way the subjects behaved during the rest of the day. Some had very large changes (i.e. muscle inactivity changed much and light muscle activity changed much) but some had modest or very little changes but this trend of substitution was systematic as indicated by the significant correlations. Thus, there is a pattern of substitution specifically between muscle inactivity and light muscle activity on a day with exercise regardless whether it was STR or RUN. The previously reported discrepancies in the associations between sedentary time and MVPA might be partly attributable to the fact that questionnaire- or accelerometer -administered time of MVPA ignores the pattern and relative intensity of the accrued MVPA. According to the hypothesis STR and RUN exercises had distinct muscle activity patterns. In STR the muscle inactivity and light and moderate-to-vigorous muscle activity all contributed about one third to the total activity profile (Figure 1). In RUN the moderate-to-vigorous muscle activity constituted over 60% of the session and light activity about 30% of the time. While muscle inactivity time during both exercises was lower than in REST, it was 80% lower in RUN than in STR in spite of the four-minute breaks in between the spurts in RUN (Table 1). Therefore, exercise sessions of seemingly similar duration can constitute of varying amount of moderate-to-vigorous muscle activity.

Further, both of the exercises resulted in increased average burst amplitudes at a similar order of magnitude despite the fact that endurance training increased moderate-to-vigorous muscle activity twice the amount of strength training. Thus, the muscle activity amplitude within the class of moderate-to-vigorous activity can vary between the training modes. Only strength training resulted in increased average muscle activity amplitude and amplitudes greater than 50% MVC over the total measurement time (Table 1–2). In this regard it is particularly important to note that the training programme was effective in increasing muscle strength during the 10-week training period. The EMG analysis showed that while the proportion of the high intensity peaks in STR session is very small (<1% of the time) they contributed to the daily activity patterns providing essential stimuli for muscle strength. This is important because larger motor units are typically recruited only at high intensities [33], which may be needed to maintain and improve neuromuscular function, and consequently to prevent muscle atrophy during aging [34]. In addition, the higher muscle activity time during RUN may result in higher metabolic demand on muscles, which may consequently increase aerobic capacity on a muscle and systemic level as suggested by the improved 3 km time trial in this study. Thus, the time spent at muscle MVPA may consist of different muscle activity patterns with different physiological effects. It is conceivable that these differences may explain part of the heterogeneous reallocation patterns between MVPA and sedentary time between studies and individuals.

In addition to the group level changes in muscle activity patterns, the type of exercise had differential effects on individual reduction of muscle inactivity. The decrease in the muscle inactivity time was rather systematic across the subjects in RUN and can be largely attributed to the increased moderate-to-vigorous muscle activity, but the amount of decrease in inactivity during STR was associated with the amount of increase in light and moderate-to-vigorous muscle activity. However, neither of the training modes influenced total daily muscle inactivity time suggesting that muscle inactivity occurring in typical sedentary activities dominates the daily activity patterns even if participants take part in well-designed exercise programmes.

These findings have two important implications. Time spent sedentary when the large muscle groups are inactive is associated with deleterious health outcomes independent of moderate-to-vigorous activity [1],[6]. This finding is supported by the distinct contraction-mediated physiological mechanisms being at play when decreasing muscle inactivity time [5], which may be partly independent of changed total energy expenditure [3],[35]. Thus, for optimal health benefits both should be targeted, and while structured training affects only MVPA during the training session, additional interventions are needed to reduce muscle inactivity time. However, the effects of reduced sedentary time are partly mediated through the activity with which it is replaced [36]. The present study showed that RUN increased moderate-to-vigorous muscle activity more than STR during the training session, suggesting that RUN may be more beneficial substitute for muscle inactivity time. Even though the effects of training were not reflected over the total measurement time, short-term physical exercise may benefit several physiological outcomes regardless of its effects on total daily energy expenditure [37].

Instead of formulating a group level hypothesis on the reallocation patterns, it would be important to find out reasons for the individual behaviour in order to improve effectiveness of interventions. For example, exercise time may simply replace sedentary time, or after having exercised people may feel more energetic towards activity outside the exercise session resulting in decreased total sedentary time [38],[39]. On the other hand, exercise may result in increased sedentary time as a compensatory mechanism to counteract the fluctuations in energy balance [40]. Yet another possibility is that exercise does not influence sedentary time because of their distinct behavioural determinants [41]. Therefore, the individual reallocation can be driven by biological or behavioural factors and should be considered depending on the primary target of a given intervention. If cardio-metabolic efficacy is targeted, the interventions should aim to dissociate the effects of sedentary time and MVPA preferably by keeping either of them or total volume of activity constant by a rigorous design [3]. On the other hand, behaviour-targeted effectiveness studies could test which of these behaviours is a more feasible target for sustained changes. It is conceivable that the different reallocation patterns may depend on age, fitness level or weight status of a participant, or environmental factors like season and affordances for physical activity [41]. In addition to studying the determinants of the individual behaviours, it would be important to study the determinants of the individual reallocation patterns in order to elucidate how a healthy total physical activity pattern could be promoted in different individuals.

The main limitation of this study is the relatively short recording time, only 9 hours for the entire day. However, the within-subject design and the similar time of day when the recordings took place counteract this limitation. Previous studies assessing habitual EMG activity have measured similar durations at similar longitudinal designs [8],[28]. Subjects who removed the shorts after evening training lack the information about evening hours that are typically abundant with sedentary behaviours [42]. While this may underestimate the amount of total daily sedentary time, the results presented as a fraction of total measurement time improve the reliability of estimated sedentary time [43], and the comparisons made in the present study remain valid. Because the recording days were pseudo-random according to availability of subjects and equipment, the fact that there were significant effects suggests that even the three days provided us sufficient data to examine the effect of strength and running training on muscle activity patterns. Some differences between the training modes may be related to the fact that EMG was measured only from the thigh muscles and upper body strength exercises were not reflected. It is possible that the thigh muscles were inactive during some upper body exercises, suggesting that the true whole body muscle exposure is underestimated in STR. The low number of subjects yielded relatively low observed power for the daily comparison (>60%) but high power for effects of exercise session (>78%). Regardless of the limitations, this study provides new information about muscle inactivity and activity patterns during both endurance and strength training exercises as well as habitual life, whereas studies using accelerometers are limited in measuring impacts without knowledge of static contractions, which may be abundant under these circumstances. Furthermore, measuring muscle inactivity periods directly from the muscles may have different physiological implications as compared to accelerometer-derived breaks which are typically averaged over 1-min epochs [6].

## 5. Conclusion

Both strength training and interval running sessions reduced muscle inactivity time and increased the time spent at muscle activities below the amplitude of 50% MVC. Only the strength training bout also increased the time spent at EMG intensities above 50% MVC. While the effects of a single bout of exercise were clear, the effects on daily muscle activity pattern were significant only for strength training bout showing increased average EMG across the entire amplitude range. However, muscle inactivity was not significantly reduced and the reallocation patterns were highly individual. The present study shows that the muscle inactivity occurring in typical sedentary activities dominates the daily activity patterns even if participants take part in well-designed exercise programmes. On the other hand, neither RUN nor STR induces compensatory increases in muscle inactivity outside of the training, but the reallocation patterns where highly individual.
